# Supplementary material for: Identification of photoperiod-induced specific miRNAs in the adrenal glands of Sunite sheep (Ovis aries)
Source: Front Vet Sci. 2022 Jul 22;9:888207. doi: 10.3389/fvets.2022.888207 (PMC9354845; doi:10.3389/fvets.2022.888207)
Supplement: Supplementary file 1 [file Data_Sheet_1.ZIP › Supplementary file/miRDeep2 file.zip!/miRDeep2 file/Novel_Structure/SP42A2/Novel_454.pdf]

Provisional ID	:	NC_019469.2_24095
Score total	:	0.1
Score for star read(s)	:	-1.3
Score for read counts	:	0
Score for mfe	:	-0.1
Score for randfold	:	1.6
Score for cons. seed	:	
Total read count	:	61
Mature read count	:	61
Loop read count	:	0
Star read count	:	0

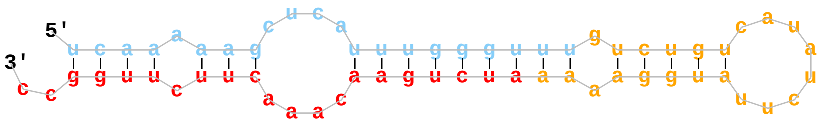

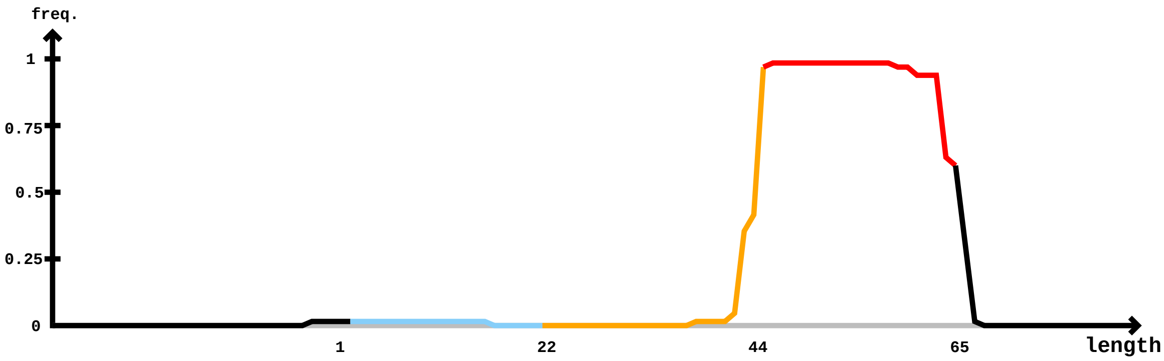


```
                                       Star                                         Mature
5'- cuggacaaaaaaagauuauauugaguuggucaaaaagcucauuuggguuugucugucauaucuuauggaaaaaucugaacaaacuucuuggccaauccagugugaaacaca   -3'    exp
    ...............((((((((.(((((((((.(((....(((((((((.(((((........))))).)))))))))....))).))))))))).))))))))......    reads      mm         sample
    .........................uuggCcaaaaagcucauuu...................................................................    1          1          seq
    .................................................................uggaaaaaucugaacaaacGu.........................    1          1          seq
    ....................................................................aaaaucugaacaaacuuUu........................    2          1          seq
    .....................................................................aaaucugaacaaacuucuugg.....................    1          0          seq
    .....................................................................aaaucugaacaaacuuUuugg.....................    19         1          seq
    ......................................................................aaucugaacaaacuuUuuggc....................    2          1          seq
    ......................................................................aaucugaacaaacuuUuuggcc...................    2          1          seq
    ........................................................................aucugaacaaacuuUuuggcc..................    25         1          seq
    ........................................................................aucugaacaaacuuUuuggcca.................    11         1          seq
    .........................................................................ucugaacaaacuuUuuggccaa................    1          1          seq
```
